# Analysis of influenza vaccination status and health information sources among middle-aged and older adults with multiple chronic diseases in Zhejiang, China: a cross-sectional study

**DOI:** 10.3389/fpubh.2025.1719412

**Published:** 2026-01-12

**Authors:** Juan Xie, Xiaotong Yan, Qi Zhang, Yue Xu, Xuehai Zhang, Dingming Yao, Jinhang Xu

**Affiliations:** 1 Zhejiang Provincial Center for Disease Control and Prevention, Hangzhou, Zhejiang, China; 2 Hangzhou Gongshu District Center for Disease Control and Prevention/Hangzhou Gongshu District Health Supervision Institution, Hangzhou, Zhejiang, China

**Keywords:** associated factors, chronic diseases, health communication, influenza vaccination coverage, multimorbidity

## Abstract

**Background:**

The growing proportion of middle-aged and older adults with multiple chronic diseases, this group faces a high risk of severe complications and death following influenza infection. However, province-level data on influenza vaccine uptake in this vulnerable group are lacking. This study assessed influenza vaccination coverage and identified factors associated with vaccination intention and behavior among multi-morbid patients in Zhejiang Province, China.

**Method:**

A convenience sampling method was utilized to select the sample. A face-to-face questionnaire survey was conducted among multi-morbid patients aged 50 and above in Zhejiang Province. Chi-square testing and multivariate logistic regression were used to analyze factors that may be associated with influenza.

**Result:**

Among 2,531 respondents, 1,390 individuals received influenza vaccination, yielding a coverage rate of 54.92%. Multivariate analysis showed age [odds ratio (OR) = 18.239, 95% confidence interval (CI): 11.718, 28.388], occupation [OR = 5.648, 95% CI: 2.935, 10.867], self-rated health status [OR = 3.070, 95% CI: 1.152, 8.179], doctor recommendation [OR = 2.586, 95% CI: 2.099, 3.186] were associated with flu vaccination. The most frequently reported reasons for vaccination were awareness of free-vaccine policies (69.85%), community campaigns highlighting vaccine benefits (54.17%), and medical advice to vaccinate (43.95%). Key reasons for non-vaccination included perceiving oneself as not at risk or vaccination as unnecessary (46.01%), doubts about vaccine effectiveness (23.14%), lack of awareness of the vaccine (22.17%), and concerns about safety or side effects (18.76%). The main sources of vaccine-related information were television (64.24%), doctors (62.62%), and family members (53.30%). Participants aged ≥80 years were more likely to obtain health knowledge from family members (χ^2^ = 8.949, *p* = 0.030). In contrast, participants aged 50–59 years were more likely to rely on WeChat (χ^2^ = 208.764, *p* < 0.001) and TikTok (χ^2^ = 191.295, *p* < 0.001) as sources of health information.

**Conclusion:**

The influenza vaccination rate among middle-aged and older adults with multiple chronic diseases in Zhejiang Province remains notably low. To enhance the willingness of this group to receive the influenza vaccine, it is essential to consider both factors that promote or hinder vaccination, along with the implementation of targeted communication strategies to effectively enhancing their willingness to receive the influenza vaccine.

## Introduction

With rapid population ageing and shifts in lifestyle patterns in China, the prevalence of chronic diseases among middle-aged and older adults has risen steadily, and the disease spectrum has increasingly shifted from single chronic conditions to multimorbidity (defined as the coexistence of two or more chronic diseases in the same individual), which has become a significant public health challenge (1, 2). Common combinations include hypertension, diabetes, coronary heart disease, chronic obstructive pulmonary disease and stroke (3). Evidence shows that the prevalence of multimorbidity increases sharply with age and is particularly common in older populations (4). International studies report multimorbidity in approximately 20–30% of the different countries, rising to 55–98% among adults aged ≥65 years (5), and exceeding 80% in those aged ≥85 years (6). For example, the prevalence of multimorbidity among older adults is about 66% in the United States and Germany (7), 75% in Australians aged >75 years (8), and 57% in Canadians aged ≥65 years (9). In China, the prevalence of multimorbidity among older adults is estimated at 55.77%, with a reported prevalence of 46.94% in Zhejiang Province (10), and is projected to continue increasing over the next two decades, underscoring the growing challenge of multimorbidity prevention and control.

Influenza is an acute respiratory infectious disease caused by influenza viruses and occurs in seasonal epidemics each year. The World Health Organization estimates that influenza causes 3–5 million cases of severe illness and 290,000–650,000 respiratory deaths globally every year (11). In China, individuals aged ≥60 years account for 80% of the nation’s influenza-associated excess respiratory mortality (12). Within Zhejiang Province, surveillance data from 2009 to 2021 indicate 990,016 reported cases and 8 deaths, with estimated actual cases 12.11-fold higher than reported (13). The Technical Guidelines for Influenza Vaccination in China (2023–2024) identify patients with chronic diseases as a key high-risk group for influenza vaccination (14). Influenza infection in individuals with chronic conditions can precipitate or exacerbate underlying diseases—for example, triggering cardiovascular events in patients with hypertension or worsening glycaemic control in patients with diabetes—thereby substantially increasing the risk of hospitalization and death (15) and imposing a considerable socioeconomic burden (16).

Compared with those with a single chronic disease, individuals with multimorbidity experience even greater clinical and economic burdens following influenza infection. One study found that patients with 2–3 chronic conditions incurred 19% higher medical expenditures, while those with 4–5 conditions had 32% higher expenditures (17). Multimorbidity often leads to atypical and overlapping clinical presentations, complex disease–disease interactions, and diagnostic challenges, which complicate treatment decision-making (18). These patients are also more prone to polypharmacy and related iatrogenic problems, including overuse of medical services. Influenza vaccination is an effective measure to prevent influenza and its complications, and has been shown to reduce influenza incidence, alleviate clinical symptoms, and decrease hospitalizations and healthcare costs among patients with chronic diseases (19–21), thereby alleviating the burden on families and society (22, 23). A meta-analysis of 95 studies confirmed vaccination prevents 28% of complications, 39% of influenza-like illnesses, and 49% of confirmed cases in this demographic (24).

However, influenza vaccination coverage among older adults with chronic diseases in China remains low, at only 0.4–5.1% (25). Although vaccination rates have increased in recent years owing to strengthened promotion efforts and the implementation of free vaccination policies for older adults (for example, 226 counties and districts offered free influenza vaccination in 2021–2022, raising local coverage in older adults to 32.9% (26)), coverage remains far below the 75% target recommended by the World Health Organization.

In the United States, European Union and other high-income settings, influenza vaccination for older adults has been incorporated into publicly funded immunization or health insurance schemes (14). By contrast, influenza vaccination for older adults in China has not yet been included in the National Immunization Program and is still primarily delivered on a voluntary, self-paid basis (27), which is an important contributor to low uptake. With ongoing economic development, several major cities such as Beijing, Shanghai and Shenzhen have introduced free influenza vaccination programs for residents aged ≥60 years (28, 29). Specific counties and districts, such as Taizhou and Ningbo, began offering complimentary influenza vaccinations to those aged ≥60 years from 2017, aligning with local government initiatives for public welfare. By 2020, Zhejiang had fully adopted the policy for residents aged ≥70 years (30), and by 2024, it was expanded to cover all residents aged ≥60 years in the province (31). Numerous studies have shown that such policies substantially increase vaccination coverage among older adults.

To date, most research on influenza vaccination in China has focused on hospitalized older adults or specific subgroups with chronic diseases, etc. To our knowledge, no studies have specifically evaluated influenza vaccination coverage and its factors among middle-aged and older adults with multimorbidity at the provincial level. Given that these individuals face a markedly higher risk of severe illness and death following influenza infection, and that the proportion of multimorbid patients within the chronic disease population continues to rise, evidence derived solely from single-disease or general older-adult cohorts is insufficient to inform precise, effective public health strategies.

Against this background, the present study aimed to: (1) estimate influenza vaccination coverage in 2024 and identify factors associated with vaccine uptake among middle-aged and older adults with multimorbidity in Zhejiang Province; (2) characterize the main sources from which this population obtains information about influenza vaccination; and (3) clarify the reasons for receiving or not receiving influenza vaccination in this high-risk group.

## Methods

### Study population

This was a cross-sectional study conducted from March to May 2024 among middle-aged and older adults with multimorbidity in Zhejiang Province. Eligible participants were patients aged ≥50 years attending hospitals at different levels who met the inclusion and exclusion criteria. Inclusion criteria were: age ≥50 years; clear consciousness and adequate communication ability; willingness to participate; and presence of multimorbidity, defined as having ≥2 physician-diagnosed chronic diseases. Exclusion criteria were: refusal to participate; or severe cognitive impairment, intellectual disability, or other conditions that precluded effective communication. The study protocol was approved by the Ethics Committee of the Zhejiang Provincial Center for Disease Control and Prevention (approval No. 2022–016-02). Written informed consent was obtained from all participants.

### Sampling strategy

**Figure 1:**
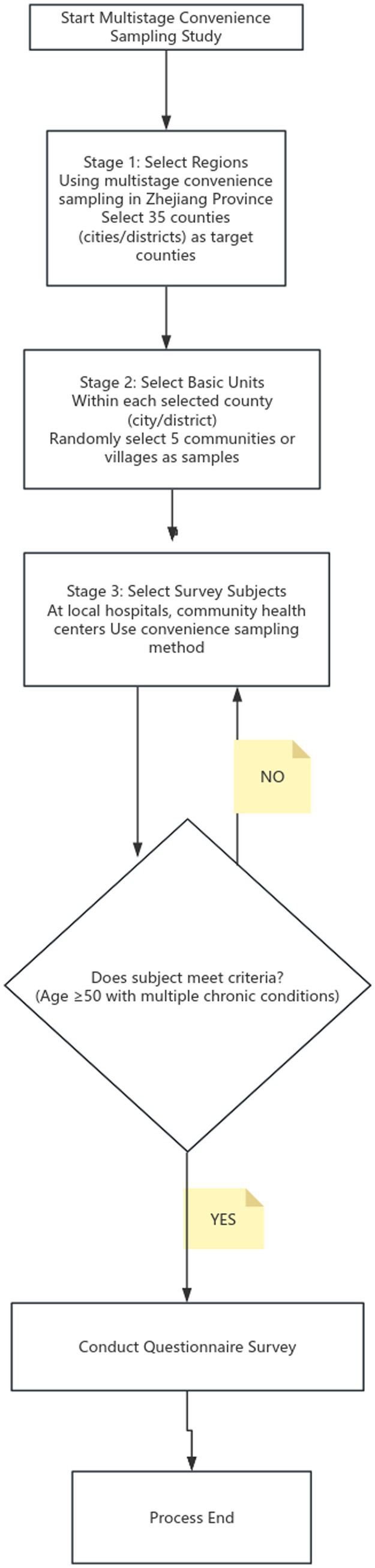
Flowchart of the multistage convenience sampling study.

A multistage convenience sampling approach was adopted. First, 35 pilot counties (cities/districts) in Zhejiang Province were selected. Second, within each selected county (city/district), 5 communities or villages were chosen as primary sampling units. Finally, in local hospitals and community health service centers/township health centers within these areas, convenience sampling was used to recruit eligible patients aged ≥50 years with multimorbidity for the survey (Figure 1).

### Data collection

Data were collected through face-to-face interviews using a structured questionnaire developed by the Health Education Institute of the Zhejiang Provincial CDC. Investigators received standardized training and passed an examination before fieldwork. A pilot survey was conducted to pretest the questionnaire, and necessary revisions were made based on expert review. The questionnaire demonstrated good reliability and validity (Cronbach’s *α* = 0.765; KMO = 0.855; Bartlett’s test, *p* < 0.01). For participants unable to complete the questionnaire independently, trained interviewers conducted the survey in an interviewer-administered format.

A standardized anonymous questionnaire designed specifically for the study was used to collect information, including sociodemographic characteristics (sex, age, marital status, household structure, educational attainment, occupation, monthly personal income, and self-rated health status),chronic disease history, influenza vaccination history, basic knowledge of influenza, reasons for vaccination or non-vaccination, health information and communication, doctor’s recommendation. The following were also taken into consideration during data collection: Chronic diseases refer to conditions that have been definitively diagnosed by a physician, including hypertension, diabetes mellitus, hyperlipidemia, chronic obstructive pulmonary disease, bronchitis, stroke, coronary artery disease, and tumors.

### Variables

Whether individuals have received the influenza vaccine is one of the dependent variables used in this analysis. This was assessed by asking respondents to answer the following statement: “Have you ever received the influenza vaccine?” Responses were categorized as “Yes” or “No.” The influenza vaccination rate was calculated as the number of individuals vaccinated against the influenza virus divided by the total number of respondents, multiplied by 100%.

The following independent variables included in the analysis were self-reported: gender (male, female); age (50–59 years, 60–69 years, 70–79 years, 80 years or above); marital status (single, married, separated/divorced/widowed); household structure type (living alone, living only with spouse, living only with children, living with spouse and children, other); education level (illiterate/semi-literate, elementary school, middle school graduation, high school graduation, university graduation or above); occupation (farmer, business personnel, medical personnel, public officials, other); personal monthly income (<2,000 RMB, 2,000–4,999 RMB, 5,000–10,000 RMB, >10,000 RMB, uncertain); self-rated health condition (good, fairly good, average, not very good, poor);and doctor’s recommendation(yes, no).

### Information channels for acquiring knowledge on vaccine immunization

Participants’ sources of vaccine administration knowledge were evaluated by asking the following question: “How did you acquire knowledge about vaccine administration? Traditional media: (1) television, (2) radio, (3) newspapers; (4) magazines/books; New media: (1) websites, (2) WeChat (3) TikTok, (4) Kwai; Interpersonal communication: (1) doctors, (2) family members, (3) friends, (4) others.” Each response option in the categories was “yes” or “no.”

### Data analysis

A database was constructed using EpiData 3.1 software (EpiData Association), with data entry executed in duplicate to ensure accuracy. We established a database for analysis with SPSS 30.0.0.0 (127). Coverage of influenza vaccination, demographics, basic knowledge of influenza, and doctor’s recommendation were analyzed descriptively. Conduct a diagnosis of collinearity among the independent variables using linear regression analysis. Chi-square testing and multivariate logistic regression were used to analyze factors that may be associated with influenza vaccination. We used a 2-tail *p*-value (*p*) significance level of 0.05.

## Results

In its entirety, the survey encompassed 2,540 participants. Among this group, 9 participants were ineligible for inclusion as they had not provided their consent. Consequently, a total of 2,531 (99.64%) participants were ultimately included in this study.

### Demographics and characteristics

Among the 2,531 participants1,324 individuals were male (52.31%) and 1,207 individuals were female (47.68%), resulting in a male-to-female ratio of 1:1.097. The majority fell within the age range of 50–79 years(90.66%),married (85.42%),living only with a spouse (48.04%),living with a spouse and children (30.81%),possessed an educational background of junior high school or below (84.98%),farmers (44.92%), enterprise personnel (28.13%), healthcare workers (3.67%), government institution staff (6.52%), reported a monthly income of less than 4,999 yuan (80.43%), self-rated physical health status as average and above (88.86%) (Table 1).

**Table 1:** Social demographic and influenza characteristics among middle-aged and older adults with multimorbidity in Zhejiang, China 2024 (*N* = 2,531).

Characteristic	Total, *n* (%)	Number of individuals vaccinated	Vaccination coverage (%)	χ^2^-value	*p*-value
Gender					
Male	1,324 (52.31)	695	52.49	6.603	0.010
Female	1,207 (47.68)	695	57.58		
Age					
50–59 years	445 (17.58)	81	18.39	490.345	< 0.001
60–69 years	840 (33.18)	370	44.05		
70–79 years	1,010 (39.9)	763	75.64		
80 years or older	236 (9.32)	175	74.04		
Marital status					
Unmarried	28 (1.1)	13	46.43	11.121	0.004
Married	2,162 (85.42)	1,162	53.75		
Separated/divorced/widowed	341 (13.47)	215	63.05		
Family structure type					
Live alone	258 (10.19)	157	60.85	28.014	< 0.001
Live only with your spouse	1,216 (48.04)	697	57.32		
Live only with their children	240 (9.48)	147	61.25		
Live with spouse and children	780 (30.81)	371	47.56		
other	37 (1.46)	18	48.65		
Degree of education					
Illiterate	466 (18.41)	310	66.52	62.622	< 0.001
Primary school complete	1,059 (41.84)	617	58.26		
Secondary school complete	626 (24.73)	285	45.53		
High school complete	256 (10.11)	118	46.09		
College complete or above	124 (4.9)	60	48.39		
Occupation^*^					
Farmer	1,137 (44.92)	702	61.74	76.739	< 0.001
Enterprise personnel	712 (28.13)	315	44.24		
Medical personnel	93 (3.67)	72	77.42		
Government institution staff	165 (6.52)	86	52.12		
Other professionals	424 (16.75)	215	50.71		
Personal monthly income					
0 to 2000	882 (34.84)	543	61.56	36.961	< 0.001
> 2000 to 4,999	1, 154 (45.59)	613	53.12		
>5,000 to 10,000	348 (13.74)	157	45.11		
>10,000	39 (1.54)	14	35.90		
Unknown	108 (4.26)	63	58.33		
Self-assessment of physical health					
Good	365 (14.42)	192	52.60	24.412	< 0.001
Relatively good	813 (32.12)	405	49.82		
Normal	1, 071 (42.32)	611	57.05		
Not very good	247 (9.76)	155	62.75		
Poor	35 (1.38)	27	77.14		
Doctor’s recommendation					
Yes	1, 861 (73.53)	1,124	80.86	85.230	< 0.001
No	670 (26.47)	266	19.14		

*If the participant has not yet reached retirement age, their occupation is recorded as their current occupation; if the participant is already retired, their occupation is recorded as their pre-retirement occupation.

### Univariate analysis of influenza vaccination

In total, 1,390 participants reported having received influenza vaccination, corresponding to a coverage rate of 54.92%. Univariable analyses showed that sex, age, marital status, household structure, educational level, occupation, monthly income, self-rated health status, and receipt of a doctor’s recommendation were all significantly associated with influenza vaccination (*p* < 0.05). Age exerted the strongest influence: vaccination coverage was highest among those aged 70–79 years (75.54%, *n* = 763) and lowest among those aged 50–59 years (18.39%, *n* = 81). Doctor recommendation was the second most influential factor: participants who reported receiving a recommendation from a doctor had markedly higher vaccination coverage (80.86%, *n* = 1,124) than those without such a recommendation (19.14%, *n* = 266). Occupation ranked third: health-care workers had substantially higher vaccination coverage (77.42%, *n* = 72) than other occupational groups (the detailed data are in Table 1, *p* < 0.05). Notably, lower educational attainment was associated with higher influenza vaccination coverage, health as poor, and participants with lower monthly income also showed higher coverage (the detailed data are in Table 1, *p* < 0.05).

### Multivariate analysis of influenza vaccination

Variables that were significant in univariable analyses were entered into a multivariable logistic regression model, with influenza vaccination status (yes/no) as the dependent variable. Collinearity diagnostics using linear regression indicated only mild collinearity among independent variables, with all variance inflation factor (VIF) values <2 (range: 1.031–1.239), suggesting minimal impact on model stability. Variables such as gender, marital status,family structure type,education, and income were not retained in the final model because they lost statistical significance (*p* > 0.05) after adjustment for other variables (e.g., occupation, self-rated health), not due to collinearity.

Binary logistic regression with forward stepwise selection identified age, occupation, self-rated health status, and doctor recommendation as independent factors associated with influenza vaccination. Compared with participants aged 50–59 years, those aged 60–69, 70–79, and ≥80 years had 4.417-, 17.809-, and 18.239- fold higher odds of being vaccinated, respectively. Health-care workers had 5.648 times higher odds of vaccination than farmers. Participants who rated their health as poor were 3.070 times more likely to be vaccinated than those who reported good health. Receiving a doctor’s recommendation was associated with 2.586-fold higher odds of influenza vaccination compared with not receiving such a recommendation (Table 2).

**Table 2:** Multivariate analysis of the impact of influenza vaccination.

Variables	β-value	S. E.	Wals	*p*-value	OR	95%*CI*
Constant	−2.107	0.537	15.371	0.000	0.122	—
Age						
50–59 years^*^						
60–69 years	1.486	0.161	85.597	<0.001	4.417	3.225 ~ 6.051
70–79 years	2.880	0.170	285.802	<0.001	17.809	12.754 ~ 24.868
80 years or older	2.904	0.226	165.473	<0.001	18.239	11.718 ~ 28.388
Occupation						
Farmer^*^						
Enterprise personnel	−0.250	0.129	3.746	0.053	0.779	0.604 ~ 1.003
Medical personnel	1.731	0.334	26.885	<0.001	5.648	2.935 ~ 10.867
Government institution staff	−0.247	0.231	1.147	0.284	0.781	0.496 ~ 1.228
Other professionals	−0.252	0.132	3.617	0.057	0.777	0.600 ~ 1.007
Self-assessment of physical health						
Good^*^						
Relatively good	−0.243	0.147	2.725	0.099	0.784	0.587 ~ 1.047
Normal	−0.110	0.143	0.588	0.443	0.896	0.676 ~ 1.187
Not very good	−0.152	0.197	0.594	0.441	0.859	0.585 ~ 1.263
Poor	1.122	0.500	5.034	0.025	3.070	1.152 ~ 8.179
Doctor’s recommendation						
Yes						
No	0.950	0.106	79.803	<0.001	2.586	2.099 ~ 3.186

* is the reference group.

### Reasons for vaccination and non-vaccination

Among the 1,390 vaccinated participants, the most commonly reported reasons for receiving influenza vaccination were awareness of local free-vaccination policies (*n* = 971, 69.85%), community campaigns emphasizing the benefits of vaccination (*n* = 753, 54.17%), recommendations from doctors (*n* = 611, 43.95%), encouragement from family members (*n* = 558, 40.14%), and a personal belief that vaccination can prevent disease (*n* = 470, 33.81%). Among the 1,141 unvaccinated participants, the leading reasons for not receiving influenza vaccination were perceiving oneself as unlikely to contract influenza or viewing vaccination as unnecessary (*n* = 525, 46.01%), doubting the effectiveness of the vaccine in preventing disease (*n* = 264, 23.14%), having never heard of the influenza vaccine (*n* = 253, 22.17%), and concerns about vaccine safety or potential side effects (*n* = 214, 18.76%) (Table 3).

**Table 3:** Factors related to the influenza vaccination of the survey participants (*N* = 2,531).

Variable	Levels	Number of respondents	Percent %
Reasons for vaccination (*n* = 1,390)	I heard there’s a free policy	971	69.85
	Community awareness of the benefits of vaccination	753	54.17
	Doctors recommend vaccination	611	43.95
	Family recommended vaccination	558	40.14
	I think vaccination can prevent diseases	470	33.81
	Everyone around has been vaccinated	373	26.83
	I’ve had the flu before, and I’ve felt the pain of it, and I want to get vaccinated in order to prevent recurrence or reduce clinical symptoms	98	7.05
Reasons for non-vaccination (*n* = 1,141)	I feel like I will not get sick, so I do not see the need to get vaccinated	525	46.01
	Even if I get vaccinated, it may not prevent the disease	264	23.14
	I have never heard of this vaccine	253	22.17
	I’m concerned that the vaccine is unsafe and will cause side effects	214	18.76
	I think vaccinations are inconvenient and cumbersome	171	14.99
	It cannot be vaccinated because of contraindications to vaccination	98	8.59
	It’s too expensive to afford	79	6.92

### Access to health information

Television, doctors, and family members emerged as the predominant channels for acquiring health knowledge. Compared with those aged 50–59, 60–69, and 70–79 years, participants aged ≥80 years were more likely to obtain health knowledge from family members (χ^2^ = 8.949, *p* = 0.030). In contrast, participants aged 50–59 years were more inclined than older age groups (60–69, 70–79, and ≥80 years) to rely on WeChat (χ^2^ = 208.764, *p* < 0.001) and TikTok (χ^2^ = 191.295, *p* < 0.001) as sources of health information (Table 4).

**Table 4:** The means of obtaining health information from different age groups of participants.

Ways of acquisition	No. of selected	Age, *n* (%)				χ^2^-value	*p*-value
		50–59	60–69	70–79	≥80		
Television	1,626 (64.24)	307 (68.99)	547 (65.12)	617 (61.09)	155(65.68)	9.229	0.026
Radio	810 (32)	184 (41.35)	252 (30)	305 (30.20)	69(29.24)	21.750	<0.001
Newspaper	589 (23.27)	140 (31.46)	192 (22.86)	209 (20.69)	48(20.34)	21.691	<0.001
Magazines/books	348 (13.75)	109 (24.49)	111 (13.21)	105 (10.40)	23(9.75)	56.293	<0.001
Websites	333 (13.16)	124 (27.87)	101 (12.02)	88 (8.71)	20(8.47)	107.185	<0.001
WeChat	842 (33.27)	247 (55.51)	337 (40.12)	221 (21.88)	37(15.68)	208.764	<0.001
TikTok	550 (21.73)	187 (42.02)	217 (25.83)	126 (12.48)	20(8.47)	191.295	<0.001
Kwai	178 (7.03)	76 (17.08)	55 (6.55)	42 (4.16)	5(2.12)	90.469	<0.001
Doctors	1,585 (62.62)	297 (66.74)	512 (60.95)	638 (63.17)	138(58.47)	6.090	0.107
Family members	1,349 (53.30)	224 (50.34)	427 (50.83)	557 (55.15)	141(59.75)	8.949	0.030
Friends	944 (37.30)	195 (43.82)	305 (36.31)	370 (36.63)	74(31.36)	12.199	0.007

## Discussion

In Middle-aged and older adults with Chronic conditions, influenza vaccination has been proven to effectively reduce hospitalization rates and mortality, thus alleviating the burden of chronic diseases (14, 32). Since 2020, Zhejiang Province has implemented a free influenza vaccination program for local residents aged ≥70 years, with planned expansion to those aged ≥60 years by 2024. In this survey, influenza vaccination coverage among multi-morbid patients was 54.92%. Although this rate is higher than that reported among older adults in Beijing and Ningbo (33, 34), it remains substantially lower than coverage levels in high-income countries such as the United States and the United Kingdom (35, 36), and falls well short of the World Health Organization (WHO) target of 75% for older adults. One plausible explanation is that, in some countries, influenza vaccination has been fully integrated into national immunization programs and is provided free of charge at the point of care. By contrast, before the introduction of the free vaccination policy in Zhejiang, the cost of vaccination had to be borne by individuals, which likely discouraged uptake, particularly among those with lower incomes. Although coverage has improved since 2020, the current level is still insufficient to establish robust population-level protection, indicating that the existing policy framework in Zhejiang Province could be further optimized.

The findings revealed that vaccination coverage was higher in those aged ≥70 years, which is consistent with the positive impact of the free vaccination policy targeting this age group (37, 38) and aligns with our finding that “awareness of free vaccination policies” was a leading reason for vaccination. Health-care workers with multimorbidity also had higher vaccination rates than other occupational groups, likely reflecting greater exposure to professional knowledge about influenza prevention and control; this pattern is consistent with findings from other domestic studies (39).

In the Health Belief Model, perceived susceptibility to disease and perceived severity of disease are two key cognitive factors that drive individuals to adopt protective behavior (40). The phenomenon observed in this study—that patients with multiple chronic diseases who rate their health as poor have a higher influenza vaccination rate—likely stems from this mechanism: individuals in poorer health have a more immediate perception of the threat posed by influenza. They are aware that their multiple chronic conditions make them more susceptible to infection (high perceived susceptibility) and are also more concerned that infection could exacerbate their existing diseases, lead to serious complications, or even death (high perceived severity). This strong perception of risk effectively offsets their concerns about the convenience or side effects of vaccination (perceived barriers), thereby significantly increasing their willingness to get vaccinated. Furthermore, in the Health Belief Model, behavioral cues are important triggers for actual action, and explicit recommendations from physicians are among the strongest of these cues (40, 41). As authoritative conveyors of health information, physicians’ recommendations can not only directly enhance patients’ perception of the benefits of vaccination but also effectively alleviate their doubts about vaccine safety and efficacy through professional explanations. This study found that the vaccination rate among those who received a physician’s recommendation was 2.586 times higher than that of those who did not—a result consistent with previous research (42), highlighting the crucial role clinicians play in promoting vaccination. In recent years, pilot programs such as the “vaccine prescription” model implemented in Zhejiang (43), Guangdong (44), and other regions have further validated the effectiveness of this mechanism in real-world promotion by strengthening physicians’ leading role in vaccine advocacy and professional guidance.

In this survey, among unvaccinated participants, the leading reasons for not being vaccinated were the belief that they were unlikely to contract influenza or that vaccination was unnecessary, doubts about vaccine effectiveness, lack of awareness of the influenza vaccine, and concerns about safety or adverse effects. These findings suggest that inadequate or distorted knowledge about influenza and influenza vaccination—particularly underestimation of disease severity and misunderstanding of the necessity of vaccination—constitutes a major barrier to uptake (45, 46). This underscores the need to strengthen targeted health education on influenza and influenza vaccination in Zhejiang Province, with a focus on correcting misconceptions, highlighting the heightened risk in individuals with multimorbidity, and emphasizing the benefits and safety of vaccination.

Health communication channels play a critical role in shaping vaccine-related perceptions and behaviours (47). In this study, television, health-care professionals, and family members were the main sources of health information for patients with multimorbidity, consistent with previous findings from China (30, 48). Traditional mass media, particularly television, remains a primary information source for middle-aged and older adults, and trust in physicians is generally high (49). Prior studies have shown that interpersonal communication can promote the adoption of health-promoting behaviours—for example, conversations about smoking cessation can enhance an individual’s intention to quit (50). Thus, beyond physician counseling in outpatient settings, strengthening health education among family members and peers may also be an effective way to encourage vaccination. Our results further revealed age-specific differences in preferred information channels. Participants aged ≥80 years relied more heavily on family members for vaccine information, whereas those aged 50–59 years were more likely than older age groups to obtain information via digital platforms such as WeChat and TikTok. With the rapid expansion of internet access in China, new media have become increasingly important for health communication. These findings suggest that future vaccination communication strategies should continue to leverage traditional media such as television—channels that middle-aged and older adults already trust—while also developing age-friendly new media approaches, such as WeChat and TikTok, to broaden the reach and visibility of vaccination information among different age groups.

This study has several limitations. First, we used a multistage convenience sampling strategy, which may have led to over-representation of individuals who are more accessible or more willing to participate, potentially overestimating vaccination willingness and affecting the representativeness and generalizability of the findings. Second, many of the interviewers were health-care workers from local hospitals or community health centres who were familiar to respondents, which may have introduced social desirability bias and further inflated reported vaccination uptake or intention. Third, key information such as influenza vaccination history and chronic disease status was self-reported and subject to recall bias, which may have affected data accuracy. In addition, the cross-sectional design precludes causal inference and limits the ability to establish temporal relationships between determinants and vaccination behavior. Finally, information sources were assessed using multiple-response items, which restricted the use of more nuanced analytical approaches (e.g., multinomial logistic regression). Future studies could consider asking respondents to rank or identify their single most important source of information to allow deeper exploration of preferences and their determinants.

In summary, influenza vaccination coverage among middle-aged and older adults with multimorbidity in Zhejiang Province remains suboptimal. To improve vaccination uptake in this high-risk group, we propose the following: (1) strengthen targeted health education on influenza and influenza vaccination for middle-aged and older adults with multimorbidity; (2) tailor communication strategies to age-specific media use patterns, combining trusted traditional media with appropriately designed new media campaigns to maximize outreach; (3) consider timely adjustments to the scope of the provincial free influenza vaccination program, such as further extending eligibility to a broader segment of high-risk older adults; and (4) enhance the role of family doctor contract services and clinical encounters, encouraging clinicians and general practitioners to proactively and systematically recommend influenza vaccination as part of routine chronic disease management and health counseling.

## Data Availability

The datasets presented in this study can be found in online repositories. The names of the repository/repositories and accession number(s) can be found in the article/Supplementary material.
