# Supplementary material for: Analysis of influenza vaccination status and health information sources among middle-aged and older adults with multiple chronic diseases in Zhejiang, China: a cross-sectional study
Source: Front Public Health. 2026 Jan 12;13:1719412. doi: 10.3389/fpubh.2025.1719412 (PMC12832235; doi:10.3389/fpubh.2025.1719412)
Supplement: Supplementary file 1 [file Table_1.docx]

| **Table 1 Social demographic and influenza characteristics among middle-aged and older adults with multimorbidity in Zhejiang, china 2024 ( N =  2,531)** | | | | | |
| --- | --- | --- | --- | --- | --- |
| **characteristic** | **Total, n (%)** | **Number of individuals**  **vaccinated** | **vaccination coverage（%）** | **χ**2***-value*** | ***P-value*** |
| **Gender** |  |  |  |  |  |
| Male | 1,324(52.31) | 695 | 52.49 | 6.603 | 0.010 |
| Female | 1,207(47.68) | 695 | 57.58 |  |  |
| **Age** |  |  |  |  |  |
| 50-59 years | 445(17.58) | 81 | 18.39 | 490.345 | < 0.001 |
| 60-69 years | 840(33.18) | 370 | 44.05 |  |  |
| 70-79 years | 1 ,010(39.9) | 763 | 75.64 |  |  |
| 80 years or older | 236(9.32) | 175 | 74.04 |  |  |
| **Marital status** |  |  |  |  |  |
| Unmarried | 28(1.1) | 13 | 46.43 | 11.121 | 0.004 |
| Married | 2 ,162(85.42) | 1,162 | 53.75 |  |  |
| Separated/divorced/widowed | 341(13.47) | 215 | 63.05 |  |  |
| **Family structure type** |  |  |  |  |  |
| Live alone | 258(10.19) | 157 | 60.85 | 28.014 | < 0.001 |
| Live only with your spouse | 1 ,216(48.04) | 697 | 57.32 |  |  |
| Live only with their children | 240(9.48) | 147 | 61.25 |  |  |
| Live with spouse and children | 780(30.81) | 371 | 47.56 |  |  |
| other | 37(1.46) | 18 | 48.65 |  |  |
| **Degree of education** |  |  |  |  |  |
| Illiterate | 466(18.41) | 310 | 66.52 | 62.622 | < 0.001 |
| Primary school complete | 1 ,059(41.84) | 617 | 58.26 |  |  |
| Secondary school complete | 626(24.73) | 285 | 45.53 |  |  |
| High school complete | 256(10.11) | 118 | 46.09 |  |  |
| College complete or above | 124(4.9) | 60 | 48.39 |  |  |
| **Occupation^*^** |  |  |  |  |  |
| Farmer | 1 ,137(44.92) | 702 | 61.74 | 76.739 | < 0.001 |
| Enterprise personnel | 712(28.13) | 315 | 44.24 |  |  |
| Medical personnel | 93(3.67) | 72 | 77.42 |  |  |
| Government institution staff | 165(6.52) | 86 | 52.12 |  |  |
| Other professionals | 424(16.75) | 215 | 50.71 |  |  |
| **Personal monthly income** |  |  |  |  |  |
| 0 to 2000 | 882(34.84) | 543 | 61.56 | 36.961 | < 0.001 |
| > 2000 to 4999 | 1, 154(45.59) | 613 | 53.12 |  |  |
| >5000 to 10000 | 348(13.74) | 157 | 45.11 |  |  |
| >10000 | 39(1.54) | 14 | 35.90 |  |  |
| Unknown | 108(4.26) | 63 | 58.33 |  |  |
| **Self-assessment of physical health** |  |  |  |  |  |
| Good | 365(14.42) | 192 | 52.60 | 24.412 | < 0.001 |
| Relatively good | 813(32.12) | 405 | 49.82 |  |  |
| Normal | 1, 071(42.32) | 611 | 57.05 |  |  |
| Not very good | 247(9.76) | 155 | 62.75 |  |  |
| Poor | 35(1.38) | 27 | 77.14 |  |  |
| **Doctor's recommendation** |  |  |  |  |  |
| Yes | 1, 861(73.53) | 1,124 | 80.86 | 85.230 | < 0.001 |
| No | 670(26.47) | 266 | 19.14 |  |  |

* If the participant has not yet reached retirement age, their occupation is recorded as their current occupation; if the participant is already retired, their occupation is recorded as their pre-retirement occupation.
