# Supplementary material for: Analysis of influenza vaccination status and health information sources among middle-aged and older adults with multiple chronic diseases in Zhejiang, China: a cross-sectional study
Source: Front Public Health. 2026 Jan 12;13:1719412. doi: 10.3389/fpubh.2025.1719412 (PMC12832235; doi:10.3389/fpubh.2025.1719412)
Supplement: Supplementary file 2 [file Table_2.docx]

| **Table 2 Multivariate analysis of the impact of influenza vaccination.** | | | | | | |
| --- | --- | --- | --- | --- | --- | --- |
| Variables | **β*-value*** | **S.E.** | **Wals** | ***P-value*** | **OR** | **95%*CI*** |
| Constant | -2.107 | 0.537 | 15.371 | 0.000 | 0.122 | — |
| **Age** |  |  |  |  |  |  |
| 50-59 years^*^ |  |  |  |  |  |  |
| 60-69 years | 1.486 | 0.161 | 85.597 | < 0.001 | 4.417 | 3.225～6.051 |
| 70-79 years | 2.880 | 0.170 | 285.802 | < 0.001 | 17.809 | 12.754～24.868 |
| 80 years or older | 2.904 | 0.226 | 165.473 | < 0.001 | 18.239 | 11.718～28.388 |
| **Occupation** |  |  |  |  |  |  |
| Farmer^*^ |  |  |  |  |  |  |
| Enterprise personnel | -0.250 | 0.129 | 3.746 | 0.053 | 0.779 | 0.604～1.003 |
| Medical personnel | 1.731 | 0.334 | 26.885 | < 0.001 | 5.648 | 2.935～10.867 |
| Government institution staff | -0.247 | 0.231 | 1.147 | 0.284 | 0.781 | 0.496～1.228 |
| Other professionals | -0.252 | 0.132 | 3.617 | 0.057 | 0.777 | 0.600～1.007 |
| **Self-assessment of physical health** |  |  |  |  |  |  |
| Good^*^ |  |  |  |  |  |  |
| Relatively good | -0.243 | 0.147 | 2.725 | 0.099 | 0.784 | 0.587～1.047 |
| Normal | -0.110 | 0.143 | 0.588 | 0.443 | 0.896 | 0.676～1.187 |
| Not very good | -0.152 | 0.197 | 0.594 | 0.441 | 0.859 | 0.585～1.263 |
| Poor | 1.122 | 0.500 | 5.034 | 0.025 | 3.070 | 1.152～8.179 |
| **Doctor's recommendation** |  |  |  |  |  |  |
| Yes |  |  |  |  |  |  |
| No | 0.950 | 0.106 | 79.803 | < 0.001 | 2.586 | 2.099～3.186 |
| * is the reference group | | | |  |  |  |
