# Supplementary material for: Analysis of influenza vaccination status and health information sources among middle-aged and older adults with multiple chronic diseases in Zhejiang, China: a cross-sectional study
Source: Front Public Health. 2026 Jan 12;13:1719412. doi: 10.3389/fpubh.2025.1719412 (PMC12832235; doi:10.3389/fpubh.2025.1719412)
Supplement: Supplementary file 3 [file Table_3.docx]

| **Table 3** **Factors related to the influenza vaccination of the survey participants（N=2,531）** | | | |
| --- | --- | --- | --- |
| **Variable** | **Levels** | **Number of**  **respondents** | **Percent**  **%** |
| Reasons for vaccination  （n = 1390） | I heard there's a free policy | 971 | 69.85 |
|  | Community awareness of the benefits of vaccination | 753 | 54.17 |
|  | Doctors recommend vaccination | 611 | 43.95 |
|  | Family recommended vaccination | 558 | 40.14 |
|  | I think vaccination can prevent diseases | 470 | 33.81 |
|  | Everyone around has been vaccinated | 373 | 26.83 |
|  | I've had the flu before, and I've felt the pain of it, and I want to get vaccinated in order to prevent recurrence or reduce clinical symptoms | 98 | 7.05 |
| Reasons for  non-vaccination  (n= 1141) | I feel like I won’t get sick, so I don’t see the need to get vaccinated | 525 | 46.01 |
|  | Even if I get vaccinated, it may not prevent the disease | 264 | 23.14 |
|  | I have never heard of this vaccine | 253 | 22.17 |
|  | I'm concerned that the vaccine is unsafe and will cause side effects | 214 | 18.76 |
|  | I think vaccinations are inconvenient and cumbersome | 171 | 14.99 |
|  | It cannot be vaccinated because of contraindications to vaccination | 98 | 8.59 |
|  | It's too expensive to afford | 79 | 6.92 |
