# Supplementary material for: Analysis of influenza vaccination status and health information sources among middle-aged and older adults with multiple chronic diseases in Zhejiang, China: a cross-sectional study
Source: Front Public Health. 2026 Jan 12;13:1719412. doi: 10.3389/fpubh.2025.1719412 (PMC12832235; doi:10.3389/fpubh.2025.1719412)
Supplement: Supplementary file 4 [file Table_4.docx]

| **Ways of acquisition** | **No. of selected** |  | **Age ,n(%)** | | | **χ**2***-value*** | ***P-value*** |
| --- | --- | --- | --- | --- | --- | --- | --- |
|  |  | **50-59** | **60-69** | **70-79** | **≥80** |  |  |
| Television | 1626（64.24） | 307(68.99) | 547(65.12) | 617(61.09) | 155(65.68) | 9.229 | 0.026 |
| Radio | 810（32） | 184(41.35) | 252(30) | 305(30.20) | 69(29.24) | 21.750 | <0.001 |
| Newspaper | 589（23.27） | 140(31.46) | 192(22.86) | 209(20.69) | 48(20.34) | 21.691 | <0.001 |
| Magazines  /books | 348（13.75） | 109(24.49) | 111(13.21) | 105(10.40) | 23(9.75) | 56.293 | <0.001 |
| Websites | 333（13.16） | 124(27.87) | 101(12.02) | 88(8.71) | 20(8.47) | 107.185 | <0.001 |
| WeChat | 842（33.27） | 247(55.51) | 337(40.12) | 221(21.88) | 37(15.68) | 208.764 | <0.001 |
| TikTok | 550（21.73） | 187(42.02) | 217(25.83) | 126(12.48) | 20(8.47) | 191.295 | <0.001 |
| Kwai | 178（7.03） | 76(17.08) | 55(6.55) | 42(4.16) | 5(2.12) | 90.469 | <0.001 |
| Doctors | 1585（62.62） | 297(66.74) | 512(60.95) | 638(63.17) | 138(58.47) | 6.090 | 0.107 |
| Family members | 1349（53.30） | 224(50.34) | 427(50.83) | 557(55.15) | 141(59.75) | 8.949 | 0.030 |
| Friends | 944（37.30） | 195(43.82) | 305(36.31) | 370（36.63） | 74（31.36） | 12.199 | 0.007 |

**Table 4.** The means of obtaining health information from different age groups of participan
